# The brave blue world: Facebook flow and Facebook Addiction Disorder (FAD)

**DOI:** 10.1371/journal.pone.0201484

**Published:** 2018-07-26

**Authors:** Julia Brailovskaia, Elke Rohmann, Hans-Werner Bierhoff, Jürgen Margraf

**Affiliations:** 1 Mental Health Research and Treatment Center, Ruhr-Universität Bochum, Bochum, Germany; 2 Department of Social Psychology, Ruhr-Universität Bochum, Bochum, Germany; Institute for Complex Systems, CNR, ITALY

## Abstract

The present study investigated the relationship between flow experienced when using Facebook (Facebook flow; i.e., experience of intensive enjoyment and pleasure generated by Facebook use due to which the Facebook activity is continued even at high costs of this behavior) and Facebook Addiction Disorder (FAD). In a sample of 398 Facebook users (age: M (SD) = 33.01 (11.23), range: 18–64), the significant positive association between Facebook flow and FAD was positively moderated by the intensity of Facebook use. Exploratory factor analysis revealed that all six items assessing FAD loaded on the same factor as two items belonging to the subscale telepresence of Facebook flow. Therefore, the close link between Facebook flow and FAD may in particular result from the immersion in an attractive online world created by Facebook, where users escape to forget their everyday obligations and problems. Present results provide first evidence that Facebook flow may be an anteceded of FAD and indicate the mechanisms that may contribute to its development and maintenance. Practical applications for future studies and limitations of present results are discussed.

## Introduction

The membership in the social networking site (SNS) Facebook brings with it many advantages (e.g., efficient communication, self-promotion, and entertainment), but may also generate some disadvantages. With respect to potential disadvantages of Facebook use, Andreassen et al. [1] investigated the so-called Facebook Addiction Disorder (FAD). They defined FAD as a subtype of behavior addictions that includes six significant characteristics, i.e., salience (i.e., permanent thinking of the SNS Facebook), tolerance (i.e., increasing amounts of Facebook use are required to achieve previous level of positive effect), mood modification (i.e., mood improvement by Facebook use), relapse (i.e., reverting to earlier use pattern after ineffective attempts to reduce Facebook use), withdrawal symptoms (i.e., becoming nervous without Facebook use), and conflict (i.e., interpersonal problems caused by intensive Facebook use). Brailovskaia and Margraf [2] demonstrated a significant increase in the number of users, who reached the critical FAD cutoff score, during a one-year period. FAD was found to be positively related to male gender, the personality traits extraversion, neuroticism and narcissism, as well as circadian rhythm (late bedtimes and rising times on weekdays and weekend). Its links to the variables age, the traits agreeableness, conscientiousness and openness, as well as physical activity were negative [2–5]. Furthermore, a positive relationship was found between FAD and the mental health variables insomnia, depression, anxiety, and stress symptoms [2, 6–8]. Additionally, recent studies reported social media addiction, which includes addictive Facebook use, to be significantly linked to different attachment styles [9] (i.e., positive: both anxious and avoidant attachment style; negative: secure attachment style), and identity styles [10] (i.e., positive: both informational and diffuse-avoidant style; negative: normative style) [11, 12]. Considering these results, the question arises which factors contribute to the development and maintenance of FAD.

Earlier studies that investigated other types of media than Facebook (e.g., video gaming, general Internet use) revealed a significant positive link between addictive behavior and flow experience [13–15]. According to the definition of Csikszentmihalyi ([16]; page 4), a flow experience is “the state in which people are so involved in an activity that nothing else seems to matter; the experience is so enjoyable that people will continue to do it even at great cost, for the sheer sake of doing it.” Some authors hypothesized that the flow experience is a positive predictor of addictive media use because the intensive enjoyment and pleasure generated by the autotelic experience, i.e., intrinsic reward, that is one of the main characteristics of flow [17], contribute to the development of a strong need to engage in excessive media use [15, 18]. Additionally, the positive link between flow and addictive media use was assumed to be strengthened by the experience of time-distortion often reported by excessive video gamers [18, 19].

Considering previous results and that Facebook use was found to be positively associated with flow experience (so-called Facebook flow) [20, 21], it seems reasonable to hypothesize that Facebook flow is positively linked to FAD and may even contribute to its development and maintenance. However, to the best to our knowledge, this link has not been investigated so far. Therefore, the main aim of the present study was to investigate whether and how Facebook flow is linked to FAD. Results may contribute to the understanding of potential risk and protective factors of the development and maintenance of FAD and may therefore be included in intervention programs to prevent Facebook addiction. This is of particular importance considering the high popularity of Facebook [22]. Facebook excels competing SNSs by far. Currently, more than two billion monthly active users are specified [23].

On the basis of this reasoning we proposed that Facebook flow and FAD are positively related (Hypothesis 1). More specifically, building on recent results (e.g., [19]), we expected to find the strongest link between the facets enjoyment and time-distortion of Facebook flow on the one hand and FAD on the other hand (Hypothesis 2). Furthermore, considering earlier findings of Wu, Scott, and Yang [15], who revealed the association between video gaming flow and addiction to be noticeable strong among experienced gamers, we assumed that the intensity of Facebook use positively moderates the link between Facebook flow and FAD (Hypothesis 3).

## Materials and methods

### Procedure and participants

Data of 398 Facebook users (73.6% women; age (years): M = 33.01, SD = 11.23, range: 18–64; occupation: 55.8% employees, 29.4% university students, 1.5% school students, 4.8% trainees for different professions like baker, 6% unemployed persons, 2.5% retirees; marital status: 29.6% single, 42.2% with romantic partner, 28.1% married) were collected from February to March 2018 via an online survey in German language. Respondents were recruited by participation invitations displayed on various SNSs (i.e., Facebook, Twitter, Xing, meinVZ). The requirement for participation, which was voluntary and not compensated, was a current Facebook membership. Although the sample is not representative of the German population in general, the participants represent diverse groups within the population as is indicated by the broad range of occupations. The use of Facebook is very popular in Germany (more than 31 million users; [24]) and its members presumably represent a cross-section of German SNSs users. Note that the participation invitation did not specify the research question neither referred to Facebook flow or FAD. Nevertheless–as in most other online studies–members, who are more active on each of the online platforms on which the participation invitation was placed, are presumably more likely to participate in the study than less active users. Research and Ethics Committee approval of the Ethics Committee of the Ruhr-Universität Bochum for the implementation of the present study was received. We followed all national regulations and laws regarding human subjects research, and obtained the required permission to conduct the present study. Participants were properly instructed and gave online informed consent to participate. The present study is part of the ongoing “Bochum Optimism and Mental Health (BOOM)” project that investigates risk and protective factors of mental health (e.g., [25]). The dataset used in the present study is available in S1 Dataset.

### Measures

#### Facebook use variables

**Facebook use intensity.** Similar to Wu, Scott, and Yang [15], to measure intensity of Facebook use, four indicators were included: duration of Facebook membership (in months), frequency of daily Facebook use, duration of daily Facebook use (in minutes), and emotional connection to Facebook and its integration into the daily life measured with the Facebook Intensity Scale (FIS; [26]). The six items of the FIS are rated on a 5-point Likert scale (1 = strongly disagree, 5 = strongly agree; e.g., “Facebook is part of my everyday activity”; earlier found internal scale reliability: Cronbach’s α = .85, current reliability: α = .82). A composite index of these four indicators was attained by computing the mean of the z-transformed indicators (α = .47).

**Facebook flow.** Flow experience related to Facebook use was assessed with a modified version of the “Facebook flow” questionnaire adopted from Kwak, Choi, and Lee [21]. After the implementation of expert reviews by three psychology trained professionals, who evaluated the appropriateness of context, conciseness, and wording of the 14 items used by Kwak, Choi, and Lee [21], eleven items divided into five subscales were selected for the present study (current reliability of the eleven items: α = .88): the subscale “focused attention” includes two items that refer to the high concentration and focus on the Facebook use; the subscale “enjoyment” consists of two items that refer to the enjoyment and pleasure/fun generated by Facebook use; the subscale “curiosity” includes two items that refer to the desire to get to know what happens on Facebook; the subscale “telepresence” consists of three items that refer to the feeling to immerse in a world created by Facebook; the subscale “time-distortion” includes two items that refer to the losing of sense of time during Facebook use. All items are rated on a 5-point Likert scale (1 = disagree strongly, 5 = agree strongly). Table 1 presents their wording and the internal reliability of the five subscales.

**Table 1:** “Facebook flow” questionnaire (modified version of [21]).

Subscales and Items	α
**FB flow subscale “Focused Attention”**	.88
1. While using Facebook, I am deeply engrossed.	
2. While using Facebook, I am immersed in the task I am performing.	
**FB flow subscale “Enjoyment”**	.90
3. Using Facebook provides me with a lot of fun.	
4. I enjoy using Facebook.	
**FB flow subscale “Curiosity”**	.70
5. Using Facebook arouses my imagination.	
6. Using Facebook excites my curiosity.	
**FB flow subscale “Telepresence”**	.84
7. Using Facebook often makes me forget where I am and what currently happens around me.	
8. Facebook creates a new world for me, and this world suddenly disappears when I stop browsing.	
9. While using Facebook, the world generated by the sites I visit is more real for me than the real world.	
**FB flow subscale “Time-Distortion”**	.79
10. Time flies when I am using Facebook.	
11. I often spend more time on Facebook than I had intended.	

FB = Facebook.

The items used in the present study are available in S2 File.

**Facebook Addiction Disorder (FAD).**The brief version of the Bergen Facebook Addiction Scale (BFAS; [1]) assessed FAD over a time frame of the last year with six items (e.g., “Felt an urge to use Facebook more and more?”) that represent the six core addiction features (i.e., salience, tolerance, mood modification, relapse, withdrawal, conflict). Items are rated on a 5-point Likert scale (1 = very rarely, 5 = very often). The BFAS has been found to exhibit similarly good psychometric properties as the full-length 18-item version (earlier reported internal reliability: α = .82-.91; e.g., [1, 3, 5, 27, 28]), as well as the Bergen Social Media Addiction Scale (BSMAS; [29]) that measures general social media addiction with six items and was derived from the BFAS (earlier reported internal reliability for the BSMAS: α = .86-.88; e.g., [11, 30]). Current reliability of the BFAS: α = .86. Two possible categorization approaches for problematic BFAS values have been suggested [1]: a more liberal approach, i.e., a polythetic scoring scheme (cutoff score: ≥ 3 on at least four of the six items), and a more conservative approach, i.e., a monothetic scoring scheme (cutoff score: ≥ 3 on all six items).

### Statistical analyses

Statistical analyses were conducted with the Statistical Package for the Social Sciences (SPSS 24) and the macro Process version 2.16.1 (www.processmacro.org/index.html).

After descriptive analyses, the associations of FAD with Facebook flow and the variables measuring Facebook use intensity were assessed by zero-order bivariate correlations. An exploratory factor analysis (EFA) using principal component analysis (PCA; rotation method: varimax) on the in total 17 items assessing Facebook flow (eleven items) and FAD (six items) was calculated. The results of the Kaiser-Meyer-Olkin (KMO = .901) and the Barlett’s test of sphericity (χ^2^ = 3856.236, df = 136, p = .000) revealed that the sample size was adequate for this analysis. Four factors had eigenvalues over 1 (factor 1: 7.322, factor 2: 2.092, factor 3: 1.199, factor 4: 1.059) and in combination explained 68.6% of the variance (factor 1: 26.3%, factor 2: 16.5%, factor 3: 14.2%, factor 4: 11.6%) (cf., [31]).

Moderation analyses (Process: model 1) examined the relationship between Facebook flow (predictor), Facebook use intensity (moderator) and FAD (outcome), controlling for age and gender as covariates. Considering the high reliability of the FIS and the low reliability of the composite index of the Facebook use intensity, two moderation analyses were run (model 1: FIS as moderator, model 2: the composite index as moderator). The moderation effect was assessed by the bootstrapping procedure (10.000 samples) that provides accelerated confidence intervals (CI 95%).

## Results

The critical cutoff score of FAD was reached by 31 (7.8%) participants following the polythetic scoring and by 15 (3.8%) participants following the monothetic scoring. Descriptive statistics of the investigated variables are shown in Table 2.

**Table 2:** Descriptive statistics of investigated variables.

	*M (SD)*	*Min–Max*
BFAS	9.49 (4.24)	6–28
BFAS: Item 1 “salience”	1.86 (1.01)	1–5
BFAS: Item 2 “tolerance”	1.73 (.99)	1–5
BFAS: Item 3 “mood modification”	1.58 (.98)	1–5
BFAS: Item 4 “relapse”	1.63 (.94)	1–5
BFAS: Item 5 “withdrawal”	1.30 (.74)	1–5
BFAS: Item 6 “conflict”	1.39 (.81)	1–5
FB flow: “Focused Attention”	2.32 (.95)	1–5
FB flow: “Enjoyment”	3.37 (.82)	1–5
FB flow: “Curiosity”	2.76 (.97)	1–5
FB flow: “Telepresence”	1.55 (.79)	1–5
FB flow: “Time-Distortion”	2.92 (1.15)	1–5
FB flow	27.41 (7.60)	11–52
FB membership (months)	83.97 (29.50)	3–155
FB visits daily (times)	11.25 (18.64)	0–200
FB use daily duration (minutes)	95.22 (81.13)	0–750
FIS	16.10 (4.98)	6–30

N = 398; M = Mean; SD = Standard Deviation; Min = Minimum; Max = Maximum; BFAS = Bergen Facebook Addiction Scale; FB = Facebook; FIS = Facebook Intensity Scale.

**Fig 1:**
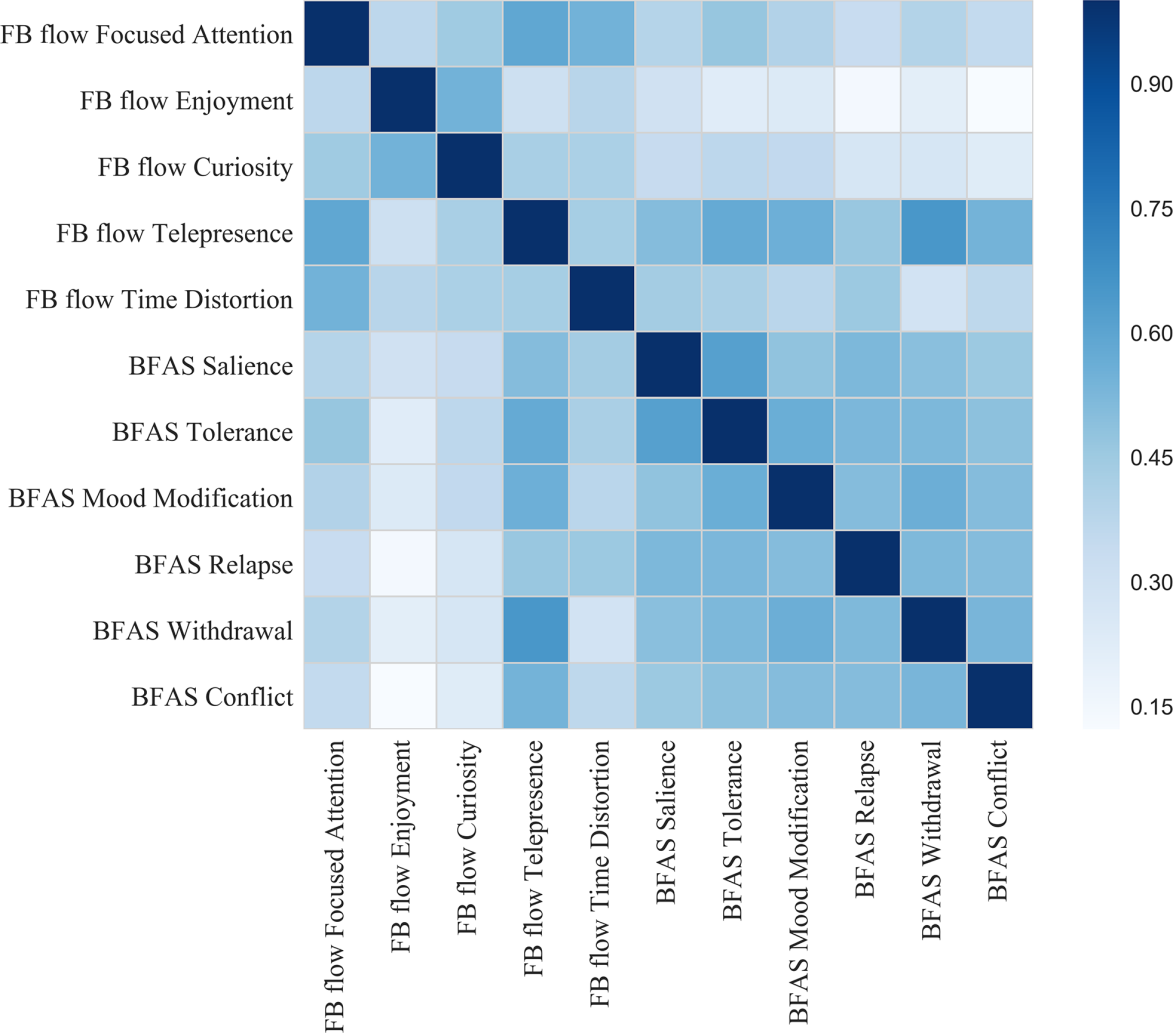
Correlogram of the correlations between the five FB flow subscales and the six FAD items (FB = Facebook; BFAS = Bergen Facebook Addiction Scale).

FAD and each of its six items were significantly positively correlated with Facebook flow and its subscales (see Table 3). Fig 1 presents a correlogram that visualizes the correlations between the five FB flow subscales and the six FAD items. In comparison with the other flow subscales, noticeable high correlations occurred for the flow subscale “telepresence”; besides the link between this subscale and FAD (r = .704, p < .001), especially its correlation with Item 5 (“withdrawal”) of FAD was high (r = .651, p < .001). Furthermore, FAD was significantly positively correlated with the four variables that represented Facebook use intensity, i.e., duration of Facebook membership, frequency and duration of daily Facebook use, and FIS (see Table 3). Also, the composite index was significantly positively related to FAD (r = .480, p < .001), as well as to Facebook flow (r = .496, p < .001).

**Table 3:** Correlations of investigated variables.

	BFAS	BFAS: Item 1 “salience”	BFAS: Item 2 “tolerance”	BFAS: Item 3 “mood modification”	BFAS: Item 4 “relapse”	BFAS: Item 5 “withdrawal”	BFAS: Item 6 “conflict”
FB flow: “Focused Attention”	.503**	.387**	.467**	.400**	.333**	.396**	.350**
FB flow: “Enjoyment”	.270**	.299**	.224**	.239**	.140**	.214**	.122*
FB flow: “Curiosity”	.398**	.339**	.369**	.355**	.268**	.267**	.226**
FB flow: “Telepresence”	.704**	.505**	.577**	.557**	.463**	.651**	.542**
FB flow: “Time-Distortion”	.509**	.435**	.420**	.374**	.456**	.290**	.364**
FB flow	.660**						
FB membership (months)	.126**						
FB visits daily (times)	.251**						
FB use daily duration (minutes)	.304**						
FIS	.513**						

N = 398; BFAS = Bergen Facebook Addiction Scale; FB = Facebook; FIS = Facebook Intensity Scale.

*p < .05

**p < .01.

The factor loadings of the rotated component matrix of the EFA show that the six FAD items and two of the three items of the subscale “telepresence” loaded all on factor 1 (factor loadings: FAD items: Item 1: .641, Item 2: .671, Item 3: .704, Item 4: .667, Item 5: .795, Item 6: .694; Facebook flow items: Item 8: .693, Item 9: .775).

**Fig 2:**
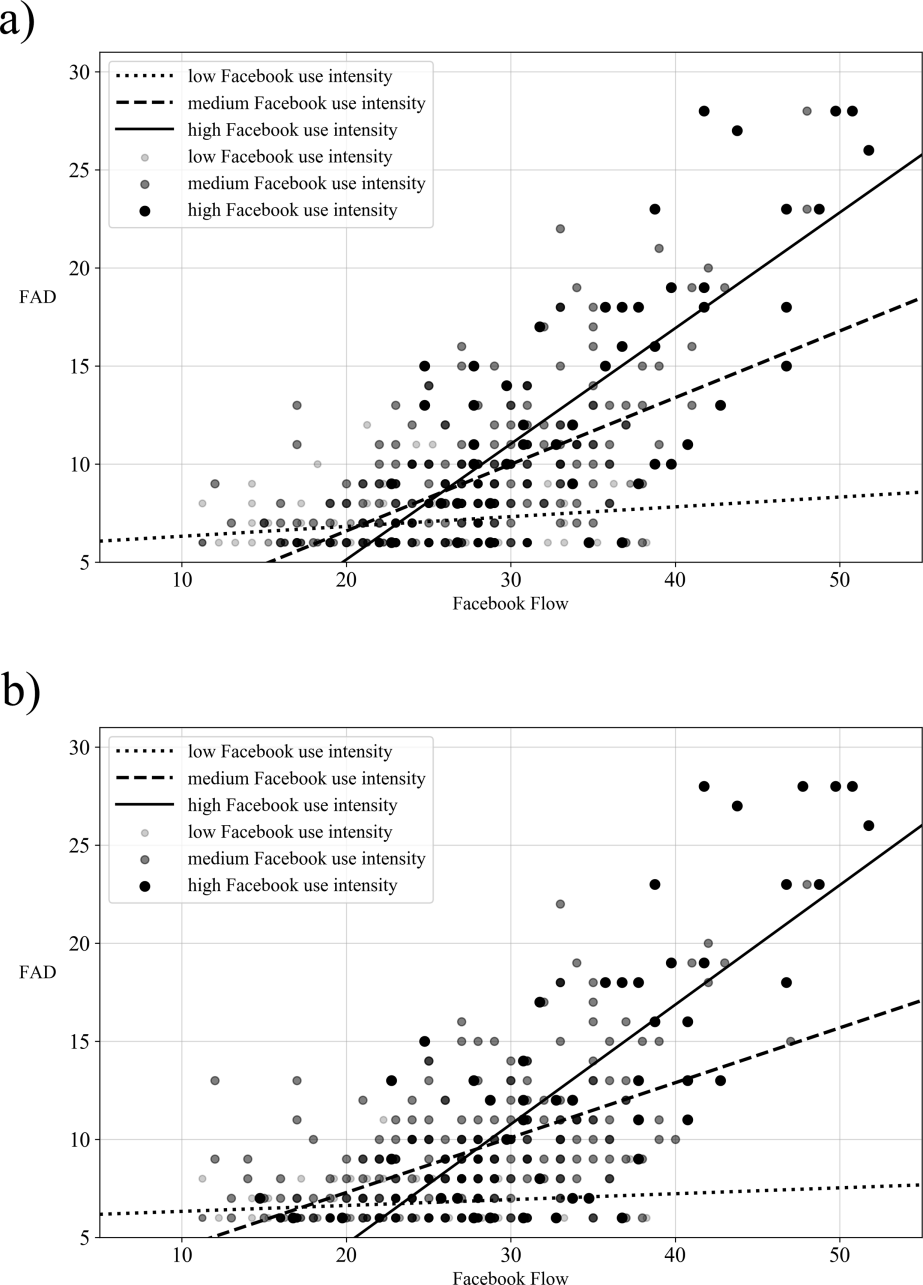
a. Moderating effect of Facebook use intensity (operationalized by Facebook Intensity Scale) on Facebook flow to FAD; b. Moderating effect of Facebook use intensity (operationalized by composite index including duration of Facebook membership, frequency of daily Facebook use, duration of daily Facebook use and Facebook Intensity Scale) on Facebook flow to FAD.

Both moderation models turned out to be statistically significant. In model 1, R^2^ = .555, F(5,392) = 54.677, p < .001, the significant interaction between Facebook use intensity (operationalized by FIS) and Facebook flow, b = .231, SE = .030, 95% CI [.173;.290], t = 7.763, p < .001, revealed that the relationship between Facebook flow and FAD was moderated by Facebook use intensity. According to the simple slopes tests, the positive link between Facebook flow and FAD was confirmed equally for low, medium, and high levels of Facebook use intensity. This link was fairly strong for participants that expressed a high level of Facebook use intensity (one SD above mean = 1.000), b = .768, SE = .066, 95% CI [.639;.897], t = 11.698, p < .001, but was weaker for participants who expressed a medium level of Facebook use intensity (mean = 0), b = .536, SE = .058, 95% CI [.423;.650], t = 9.287, p < .001, and noticeable weaker for those participants with a low level of Facebook use intensity (one SD below mean = -1.000), b = .305, SE = .064, 95% CI [.178;.431], t = 4.738, p < .001 (see Fig 2, part a).

Fig 2 (part b) presents model 2, R^2^ = .566, F(5,392) = 54.786, p < .001. As revealed by the significant interaction between Facebook use intensity (operationalized by the composite index) and Facebook flow, b = .345, SE = .053, 95% CI [.241;.449], t = 6.506, p < .001, the relationship between Facebook flow and FAD was moderated by Facebook use intensity. Again, the simple slopes tests showed that the positive link between Facebook flow and FAD was confirmed equally for low, medium, and high levels of Facebook use intensity. It was fairly strong for participants that expressed a high level of Facebook use intensity (one SD above mean = .622), b = .728, SE = .059, 95% CI [.612;.843], t = 12.347, p < .001, but was weaker for participants who expressed a medium level of Facebook use intensity (mean = 0), b = .513, SE = .048, 95% CI [.419;.607], t = 10.711, p < .001, and noticeable weaker for those participants with a low level of Facebook use intensity (one SD below mean = -.622), b = .298, SE = .057, 95% CI [.185;.411], t = 5.196, p < .001 (see Fig 2, part b).

## Discussion

The present study investigated the link between flow experienced on the SNS Facebook and FAD. In line with earlier studies that described flow experience and addictive media use to be positively interrelated [15, 18, 19], current findings revealed a significant positive association between Facebook flow and FAD (confirming Hypothesis 1). Note, that the link was considerably strong as the common variance between both variables was 43.6%. Also, each subscale of Facebook flow was significantly positively related to FAD. However, in contrast to our expectations that rested on previous results (e.g., [18]), the subscales enjoyment and time-distortion of Facebook flow did not show the strongest link with FAD. The link with the scale “enjoyment” was the weakest one of the five flow subscales (contradicting Hypothesis 2). In comparison, the highest correlation emerged between FAD and the subscale “telepresence” (effect size of the correlation differences Cohen’s q ranges from .31 to .60; cf., [32]). In particular the FAD item “withdrawal” was closely linked to this subscale. Furthermore, all six items assessing FAD loaded on the same factor as two items of the scale “telepresence”.

The subscale “telepresence” measures the feeling to immerse in a world created by Facebook [21]. While the two items of this subscale (Item 8 “Facebook creates a new world for me, and this world suddenly disappears when I stop browsing”, Item 9 “While using Facebook, the world generated by the sites I visit is more real for me than the real world”), which loaded on the same factor as the FAD items, included the immersion in a new world in wording, this was not the case for the third item (Item 7 “Using Facebook often makes me forget where I am and what currently happens around me”), which loaded on another factor. Earlier research identified telepresence to be one of the main factors that causes flow experienced in the online environment [33]. The more lifelike images the appropriate online environment includes, the more immersed users feel in it [34, 35]. Facebook members daily upload millions of private photos to share their experiences with their online friends and to involve them in their life [22, 23]. Therefore, they contribute to the permanent development of the Facebook world, which opens its members different ways of (social) interaction. Some Facebook members, in particular those who score high on depression and anxiety symptoms, pursue this interaction for escape from daily problems and to elicit positive experiences often missed offline [6]. Furthermore, it should be considered that earlier research reported a positive association between narcissism and FAD [2]. Individuals high on narcissism, who are characterized by an inflated sense of entitlement and conviction of own grandiosity, typically search intensively for attention and admiration. When they are unable to get this positive feedback, or perceive information which contradicts their inflated self-view, their self-esteem suffers [36, 37]. Thus, it can be hypothesized that narcissistic people, also prefer to escape from daily problems by excessively using Facebook, whereby the probability to get a lot of positive feedback, e.g., “Likes” or positive comments, from a large audience in a short period of time is often remarkably higher than it is the case of interaction in the offline world”.

Considering our current results, these individuals might be at special risk to develop FAD. When the immersion in the Facebook world causes an intensive intrinsic reward, the probability that Facebook will be employed more excessively increases. However, according to present findings that confirmed our Hypothesis 3, Facebook use intensity, assessed either by FIS or by the composite index, positively moderates the relationship between Facebook flow and FAD. Especially members who intensively use Facebook, i.e., frequently visit it, spend there a lot of time, integrate Facebook use in their daily life, and develop an emotional connection to it, seem to experience high values of Facebook flow and are particularly prone to FAD. It can be hypothesized that an additional risk factor to develop FAD occurs when the overlap between the offline and online relationships is small and the amount of online relationships considerably outweighs that of the offline relationships. This constellation contributes to the development of a strong emotional attachment to Facebook [38], which is supposed to increase the impact of the telepresence of the online world on the individual. In the extreme case, the immersion in the online world might become so intensive that the affected individual cannot recognize anymore the difference between the online and offline world. Considering the close link between attachment styles and addictive social media use reported in recent studies [11, 12], the conclusion is justified that the risk for the development of a strong attachment to Facebook is especially high for Facebook members with an anxious attachment style, who often engage in excessive social media use to satisfy their need for approval and positive feedback. In contrast, Facebook users who exhibit a secure attachment style may be less prone to this risk.

Present findings are of particular importance because they reveal that Facebook flow in general and the telepresence experienced on Facebook in particular might contribute to the development and maintenance of FAD. FAD indications occurred in 3.8% (monothetic scoring) to 7.8% (polythetic scoring) of our sample, which because of its higher age and occupation range (70.6% non-students) is more representative of the general population than samples of earlier studies on FAD, which mostly included undergraduate students only (e.g., [2, 4, 7, 27, 28]). Considering rates of FAD indications and the relatively high representativeness of present sample the conclusion is justified that FAD no longer constitutes a negligible marginal phenomenon. Thus, it might be effective to apply present findings on intervention programs against addictive media use. One suggestion would be to emphasize the desirability to consciously regulate the Facebook use intensity by for example setting clear time limits for daily use. In addition, in earlier studies on addictive video gaming and problematic general Internet use [18, 39], it has been suggested to deploy an alarm clock or to include “pop-up” messages to regulate usage time. These procedures are likely to be supportive in preventing excessive Facebook use that increases vulnerability to FAD. Furthermore, it is important to raise awareness of the fact that the Facebook world even if it is employed to stay connected with offline friends and family members still remains a virtual space and that the escape into the online world mostly does not contribute to problem-solving offline. In contrast, excessive Facebook use may contribute to the aggravation of existing problems or result in the emergence of new problems. For example, 11.1% of the current sample indicated to use Facebook so much that it has had a negative impact on their job/studies (FAD Item 6 “conflict”).

Although the current study has many assets and may contribute to an improvement of intervention programs of addictive media use, some of its limitations are worth mentioning. The most important weakness is its cross-sectional design which allows only limited conclusions with respect to causality [40]. Although it is quite plausible that Facebook flow causes FAD (and not vice versa) and that the moderating influence of Facebook use intensity corresponds with such a causal structure, this reasoning is hypothetical. Therefore, we strongly advise future researchers to consider the link between Facebook flow and FAD by longitudinal prospective designs and by experimental research.

Additionally, the gender composition (73.6% female) of our sample limits the generalization of current results. To tackle this limitation, we controlled for the variable gender in our statistical analyses. Nevertheless, it is desirable to replicate the current results in a sample with an equal gender ratio to enable more generalizable conclusions.

Furthermore, it should be considered that participants of the current study were recruited by participation invitations displayed on different online SNSs. Thus, it cannot be excluded that the more a user was active on the appropriate online platform, the higher was the probability that this user became aware of the invitation and responded to the offer to participate. Moreover, due to the voluntary nature of the participation, it could be that particularly individuals, who were already interested in online research on SNSs responded to the online survey. This potential selection bias limits the generalizability of current results. It is likely that regular users of SNSs participated more likely in the study than infrequent users. This bias, which is common in many online studies, could result in a range restriction of the sample in terms of amount of SNSs use. Although such a range restriction might possibly have reduced the magnitude of correlations involving Facebook flow and FAD, it is unlikely that it threated the validity of current statistical analyses. The hypotheses tests turned out to be significant indicating that potential range restrictions did not reduce considerably the sensitivity of the statistical tests performed. In addition, it is likely that the specific research question of the study did not influence the decision to participate in the study because it was not revealed in advance to the participants.

To sum up, the present study reveals a close positive interplay between Facebook flow and FAD. In particular the telepresence of the Facebook world, which is an important characteristic of Facebook flow, seems to enhance the individual vulnerability to develop FAD. The interplay between Facebook flow and FAD should be further investigated to better understand the risk of the development of FAD as well as the role of protective factors against it.

## Supporting information

S1 File Dataset used for analyses in present study. (SAV)

S2 File Used items. (DOCX)
