# Supplementary material for: The brave blue world: Facebook flow and Facebook Addiction Disorder (FAD)
Source: PLoS One. 2018 Jul 26;13(7):e0201484. doi: 10.1371/journal.pone.0201484 (PMC6062136; doi:10.1371/journal.pone.0201484)
Supplement: S2 File — (DOCX) [file pone.0201484.s002.docx]

**Age (in years):** ___________

**Gender:** women men

**Marital status**

| Single | **🞏1** |
| --- | --- |
| With romantic partner | **🞏2** |
| Married | **🞏3** |

**Current occupation:**

University student

Trainee

Employee

Unemployed

Retiree

School student

**For how long are you a Facebook member?** _____ months

**How often do you use Facebook daily?** _____ times

**How much time do you spend in Facebook daily?** _____ minutes

|  |  | 1 = strongly disagree | 2 = disagree | | 3 = neutral | 4 =  agree | 5 =  strongly agree |
| --- | --- | --- | --- | --- | --- | --- | --- |
| 1 | Facebook is part of my everyday activity. | 🞏 | 🞏 | 🞏 | | 🞏 | 🞏 |
| 2 | I am proud to tell people I'm on Facebook. | 🞏 | 🞏 | 🞏 | | 🞏 | 🞏 |
| 3 | Facebook has become part of my daily routine. | 🞏 | 🞏 | 🞏 | | 🞏 | 🞏 |
| 4 | I feel out of touch when I haven't logged onto Facebook for a while. | 🞏 | 🞏 | 🞏 | | 🞏 | 🞏 |
| 5 | I feel I am part of the Facebook community. | 🞏 | 🞏 | 🞏 | | 🞏 | 🞏 |
| 6 | I would be sorry if Facebook shut down. | 🞏 | 🞏 | 🞏 | | 🞏 | 🞏 |

|  |  | 1 = strongly disagree | 2 = disagree | 3 = neutral | 4 =  agree | 5 =  strongly agree |
| --- | --- | --- | --- | --- | --- | --- |
| 1 | While using Facebook, I am deeply engrossed. | 🞏 | 🞏 | 🞏 | 🞏 | 🞏 |
| 2 | While using Facebook, I am immersed in the task I am performing. | 🞏 | 🞏 | 🞏 | 🞏 | 🞏 |
| 3 | Using Facebook provides me with a lot of fun. | 🞏 | 🞏 | 🞏 | 🞏 | 🞏 |
| 4 | I enjoy using Facebook. | 🞏 | 🞏 | 🞏 | 🞏 | 🞏 |
| 5 | Using Facebook arouses my imagination. | 🞏 | 🞏 | 🞏 | 🞏 | 🞏 |
| 6 | Using Facebook excites my curiosity. | 🞏 | 🞏 | 🞏 | 🞏 | 🞏 |
| 7 | Using Facebook often makes me forget where I am and what currently happens around me. | 🞏 | 🞏 | 🞏 | 🞏 | 🞏 |
| 8 | Facebook creates a new world for me, and this world suddenly disappears when I stop browsing. | 🞏 | 🞏 | 🞏 | 🞏 | 🞏 |
| 9 | While using Facebook, the world generated by the sites I visit is more real for me than the real world. | 🞏 | 🞏 | 🞏 | 🞏 | 🞏 |
| 10 | Time flies when I am using Facebook. | 🞏 | 🞏 | 🞏 | 🞏 | 🞏 |
| 11 | I often spend more time on Facebook than I had intended. | 🞏 | 🞏 | 🞏 | 🞏 | 🞏 |

|  | How often during the last year have you… | 1=  very rarely | 2=  rarely | 3=  sometimes | 4=  often | 5=  very often |
| --- | --- | --- | --- | --- | --- | --- |
| 1 | …spent a lot of time thinking about Facebook or planned use of Facebook? | 🞏 | 🞏 | 🞏 | 🞏 | 🞏 |
| 2 | ...felt an urge to use Facebook more and more? | 🞏 | 🞏 | 🞏 | 🞏 | 🞏 |
| 3 | ...used Facebook in order to forget about personal problems? | 🞏 | 🞏 | 🞏 | 🞏 | 🞏 |
| 4 | ...tried to cut down on the use of Facebook without success? | 🞏 | 🞏 | 🞏 | 🞏 | 🞏 |
| 5 | ...become restless or troubled if you have been prohibited from using Facebook? | 🞏 | 🞏 | 🞏 | 🞏 | 🞏 |
| 6 | ...used Facebook so much that it has had a negative impact on your job/studies? | 🞏 | 🞏 | 🞏 | 🞏 | 🞏 |
